# Water-Resistant Mechanoluminescent Electrospun Fabrics with Protected Sensitivity in Wet Condition via Plasma-Enhanced Chemical Vapor Deposition Process

**DOI:** 10.3390/polym12081720

**Published:** 2020-07-31

**Authors:** Halim Lee, Eunjin Cho, Tomas Webbe Kerekes, Seung Lee Kwon, Gun Jin Yun, Jooyoun Kim

**Affiliations:** 1 Department of Textiles, Merchandising and Fashion Design, Seoul National University, Seoul 08826, Korea; hlee335@snu.ac.kr (H.L.); kkumdal98@snu.ac.kr (E.C.); 2 Department of Mechanical and Aerospace Engineering, Seoul National University, Seoul 08826, Korea; tomaswebbe@gmail.com (T.W.K.); kwonsl7912@gmail.com (S.L.K.); 3 Institute of Advanced Aerospace Technology, Seoul National University, Seoul 08826, Korea; 4 Research Institute of Human Ecology, Seoul National University, Seoul 08826, Korea

**Keywords:** mechanoluminescence, electrospinning, water-resistant, plasma-enhanced chemical vapor deposition, sensor, smart textile

## Abstract

Mechanoluminescence (ML), which emits light upon external mechanical stress, was applied to fibrous composites. Herein, ML particles were incorporated into poly(vinylidene fluoride) (PVDF) and polyacrylonitrile (PAN) electrospun webs to prepare ML/PVDF and ML/PAN composite fabrics. The produced fabrics were treated with O_2_ and C_4_F_8_ plasma to modify the wetting properties, then the effects of composite wettability on the light-emitting response in dry and wet conditions were investigated. The light intensity was greatly decreased when the composite fabrics absorbed water. When the composites were hydrophobized by the C_4_F_8_ plasma-enhanced chemical vapor deposition process, the original light intensity was protected in wet conditions, while maintaining the water vapor transmission rate. As the clothing material would be exposed to moisture in varied situations, the reduced ML sensitivity in wet conditions may limit the application of ML composite fabrics. The findings suggest a facile strategy to fabricate moisture-resistant, breathable mechanoluminescence composite fabrics.

## 1. Introduction

The vigorous development of smart textiles has led to a new era of lifestyle. Countless smart textiles or devices are capable of monitoring and sensing natural phenomena around everyday life [1]. Most current smart textiles are powered by traditional rechargeable batteries which are large, heavy, and bulky; therefore, they are unsuitable to properly integrate with textiles, making the consolidation of a single system infeasible [2]. For this reason, there is a special interest to develop pliable and lightweight alternatives for electrical power generation and storage, such as flexible and elastic batteries [3,4,5], supercapacitors [6], photovoltaic [7], thermoelectric [8], and piezoelectric generators [9,10]. One way to fulfill the demands of mechanical sensors that work without batteries is by using mechanoluminescence. The development of mechanoluminescent (ML) materials has contributed to a broad source of multi-functional components with a wide range of applications in diverse fields [11,12]. It is well known that ML materials emit light when subjected to mechanical stress such as elastic–plastic deformation or even friction [13,14].

ML emits light according to a band theory, in which an electron gains energy from the external mechanical stress, where it goes to an excited state and then it releases the light when coming back to the ground state [15]. A distinct characteristic of ML appealed to researchers in materials science, leading to a wide scope of ML research [16]. Various types of ML with different components such as SrAl_2_O_4_:(Eu^2+^, Dy^3+^), ZnS:Mn, Ca_2_MgSi_2_O_7_:Eu, and CaZnOs:Mn have been observed and the investigation is still ongoing [17]. Among the earlier findings of ML, the material used in the experiment was green-emitting phosphor of SrAl_2_O_4_:Eu^2+^ co-doped with Dy^3+^ (SAOED) which has been known as intense and long-lasting phosphorescence [18,19]. Recently, ML materials have caught the attention of researchers for application in a wide range of nondestructive evaluations [20,21,22] and for diverse types of mechano-optical devices [23,24]. The investigation has been conducted for the measurement and visualization of stress distribution of the SAOED-incorporated solids in the form of ML paint [25,26]; ML sensing film and adhesive [11,27,28,29]; and ML/epoxy composites [13,30,31,32].

When ML materials containing alkaline earth aluminates interact with water, the properties of both phosphorescence and mechanoluminescence can be deteriorated as the hydrolysis reaction of ML occurs. As clothing textiles can be exposed to the moist and sweaty environment, hydrolysis of ML in wet conditions puts a significant constraint on ML application to textiles. To prevent the hydrolysis of ML materials, several studies suggested introducing a water-resistant protection barrier for ML particles [33,34,35]. Such barrier materials often involve a complicated chemical procedure [35,36], and loss of flexibility and permeability, limiting the application to textile materials.

Herein, water-resistant yet breathable nonwoven fabrics with mechanoluminescence performance were proposed to detect mechanical stimulation, in consideration of the practical application in wet conditions. For this purpose, ML composite fabrics were fabricated by electrospinning of ML-incorporated PVDF and PAN fibers. The electrospinning technology used in this paper has a wide range of applications, which allows us to fabricate nanomaterials with little restriction on materials. With versatile process options, electrospinning is extending its capability of creating novel functional nonwovens [37,38] and composite mats by combining functional materials with polymers [39,40]. This study employed a simple electrospinning process by dispersing the ML particles in the pre-spinning polymer solution to produce particle-incorporated composite webs.

The ML-incorporated PVDF electrospun web (ML/PVDF) and ML-incorporated PAN electrospun web (ML/PAN) were further modified to vary in wetting properties, either being hydrophilic or hydrophobic via a simple plasma process. The as-spun hydrophilized and hydrophobized composite fabrics of ML/PVDF and ML/PAN were compared for water add-on (%) and wettability. The composites with different wettability were investigated for the ML performance both in dry and wet states. The breathability of ML composite fabrics was examined by measuring the water vapor transmission rate. In consideration of the textile application, the effects of composite thickness and layering on ML performance were investigated, and simulation was also conducted to interpret the results.

Ultimately, the study intends to suggest a facile strategy to fabricate the breathable composite fabrics of which performance is well maintained in wet conditions. The approach of this study is novel in that a simple technique of the plasma-enhanced chemical vapor deposition (PECVD) process was applied to composite fabrics to optimize ML performance in wet conditions. As PECVD produced a very thin layer of coating, the breathability of the composite fabric was maintained. The resulting material can be applied as a battery-free mechanoluminescent sensor in smart textiles.

## 2. Materials and Methods

### 2.1. Materials

Mechanoluminescence (strontium aluminate co-doped with europium ions and dysprosium ions, SrAl_2_O_4_:(Eu^2+^, Dy^3+^)) particles were purchased from Nemoto & Co (Tokyo, Japan). Poly(vinylidene fluoride) (PVDF) resin (*M*_w_ 275,000) and polyacrylonitrile (PAN) resin (*M*_w_ 150,000) were purchased from Sigma-Aldrich (St. Louis, MO, USA). N, N-dimethylformamide (DMF) and acetone were bought from Daejung Chemicals (Siheung-si, Gyeonggi-do, South Korea). Calcium chloride, anhydrous, 96.0% was purchased from Samchun Chemicals (Seoul, South Korea). Octafluorocyclobutane (C_4_F_8_) gas and oxygen gas (O_2_) were purchased from Union Gas (Yongin-si, Gyeonggi-do, South Korea).

### 2.2. Fabrication of ML/PVDF and ML/PAN

PVDF pellets were dissolved in a blend of (1:1 volume ratio) DMF and acetone, and the PVDF pre-spinning solution was prepared in 26% (*w*/*v*). ML powder was added to the respective pre-spinning solutions with 7.8% (*w*/*v*) of the solution. The ML in the pre-spinning solution was mixed in a vial using a magnetic stir bar at 60 °C for 24 h. The ML-containing pre-spinning solution was electrospun at the bias voltage of 10~12 kV and the feeding rate of 4 mL/h. The electrospun fibers were collected to a rotating drum collector wrapped with paper foil (180 rpm) at a distance of 15 cm. The fiber was directly collected on the paper foil. The collected electrospun composite web was dried in an oven at 40 °C for 24 h. The thickness of the PVDF electrospun web was varied to 0.1, 0.2, and 0.4 mm.

Polyacrylonitrile resin was mixed with DMF with 10% (*w*/*v*) to make a PAN pre-spinning solution. The ML was added to the pre-spinning solution in 7.8% (*w*/*v*) of the solution. The *w/v*% of ML in the composite web was adjusted to be the same for the ML/PVDF and ML/PAN webs. The ML-containing PAN solution was electrospun at 15~18 kV with a 1 mL/h feeding rate. The fibers were collected onto a rotating drum collector (at 200 rpm) from a distance of 15 cm. The electrospinning process parameters were adjusted to produce beadless fibers in the consistent size range for the respective fibers, without clogging of the nozzle. Electrospinning was conducted under a temperature of 20 ± 2 °C and relative humidity of 20 ± 3% RH.

The surface chemistry of ML-incorporated electrospun fabric was modified by the plasma process. To attach the oxygen group to the composite surface, the composite fabric was subject to an O_2_ plasma treatment for 5 min at 200 W with 160 cm^3^/min in the plasma system (COVANCE, FemtoScience, Hwaseong, South Korea) [41]. To coat the surface with the fluorinated compound by the plasma-enhanced chemical vapor deposition (PECVD), the composite fabric was treated under C_4_F_8_ gas for 25 min at 200 W with 100 cm^3^/min [42,43]. The generated frequency of plasma was 50 kHz in both O_2_ and C_4_F_8_ plasma processes.

The wettability of the composite surface was examined via measurement of the static contact angles (CA) of water, using an optical tensiometer (Theta Lite, KSV Instruments Ltd., Espoo, Finland). For the CA measurement, a 3.4 µl of water drop was placed on a surface, and the CA was measured within 5 secs after the deposition of a liquid drop. The measurement was done on at least five different locations of the sample surface.

### 2.3. Measurement of Mechanoluminescence by the Ball Drop Test

**Figure 1:**
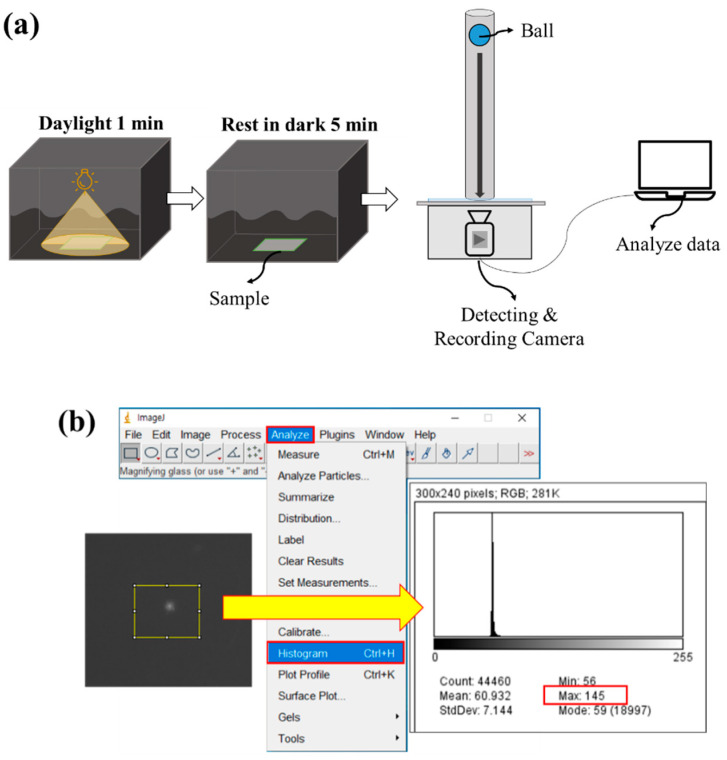
Light emission investigation of the mechanoluminescence (ML)/poly(vinylidene fluoride) (PVDF) composite. (**a**) Test setup for measurement of mechanoluminescence of ML composite fabrics; (**b**) image processing after drop ball test using ImageJ.

To measure the intensity of ML of the composites, a ball drop test was designed, where a 23 g spherical glass ball with a 2.5 cm radius was vertically dropped from a 20 cm distance from the composite surface (Figure 1a). The light intensity was measured when the fabric experienced instantaneous stress. For charging the photo energy to the samples, the composite samples were rested in the daylight condition for 1 min using standard lighting equipment (CO−204, Hanwon Soway Co., Seoul, South Korea). After 1 min, the samples were rested in the darkroom for 5 min to discharge photo energy to control the maximum pixel intensity value (PIV) which reflects the light intensity of the sample. The maximum measurable PIV (unitless value) was 255, and the minimum was 0. Both charging time (1 min) and discharging time (5 min) were maintained the same to control the light intensity of ML composites.

A Grasshopper 3 4.1MP^®^ (FLIR Systems, Inc., Wilsonville, OR, USA) camera was used in conjunction with the PTGrey Fly Capture 2^®^ (FLIR Systems, Inc.) software for video recording the moment of the impact. Shutter speed, aperture, and focus were adjusted to collect the best possible image definition; these settings remained unaltered for all tests. The images were evaluated using the image processing software FIJI ImageJ2^®^ [44,45], applying a greyscale range for measuring the PIV. The same detection procedure was applied in previous studies [46,47,48,49]. In this method, the PIV was calculated on the grayscale and the sum of the gray value in the selected area was divided by the number of pixels. As the light gets brighter, the PIV presents a higher value. The maximum PIV of the responding area is obtained for the analysis (Figure 1b) because the light intensity of the ML/polymer composite is focused on a micro point of view. The ball drop test setup and the image process are shown in Figure 1.

### 2.4. Microscopic Analysis

The fluorescence image of the composite fabrics was observed by the fluorescence microscope (U-HGLGPS, Olympus, Tokyo, Japan). The surface morphology and ML size were observed using a field-emission scanning electron microscope (FE-SEM, MERLIN Compact, Carl Zeiss, Jena, Germany), with a prior Pt coating (~10 nm) at 10 mA for 180 s using a sputter coater (EM ACE200, Leica, Wetzlar, Germany). Energy-dispersive spectroscopy (EDS) analysis and elemental mapping were achieved by a NORAN system 7 attached to the SEM equipment. 

### 2.5. Measurement of Water Vapor Transmission Rate (WVTR)

The water vapor transmission rate (WVTR) of the composite fabric was measured following KS K 0594:2015 testing methods for the water vapor transmission rate of textile fabrics [50]. A combined temperature and humidity chamber (PL—3KPH, Espec Corp., Osaka, Japan) was used. The thickness of the ML/PVDF specimen used for the experiment was 0.1 ± 0.02 mm and ML/PAN was 0.13 ± 0.02 mm. A composite sample with a 7 cm diameter was fixed on a water-permeable cup containing 33 g of calcium chloride (CaCl_2_) at 40 ± 2 °C, 90 ± 5% RH condition. The cup with a composite sample must maintain a 3 mm distance between the sample and the CaCl_2_. After 1 h, the weight (*a*_1_) of the specimen is measured immediately. Then, put the test specimen back into the chamber and take out the specimen again to measure the weight (*a*_2_) after the following 1 h. The mass change (g) was measured after a predetermined time to calculate the WVTR by the following equation.

(1) $$P=\frac{a_{2}\;-\;\;a_{1}}{S}$$

*P*: water vapor transmission rate (g/m^2^·h);
*a*_1_–*a*_2_: mass change of water-permeable cup with CaCl_2_ after 1 h (g/h);
*S*: area (m^2^) of the sample exposed to the moisture absorbent.

### 2.6. Finite Element Analysis for Normal Stress

Abaqus/Explicit [51] was utilized for a drop ball test simulation. The ML composite material is assumed to be linearly elastic and its properties used in the simulation are listed in Table 1.

**Figure 2:**
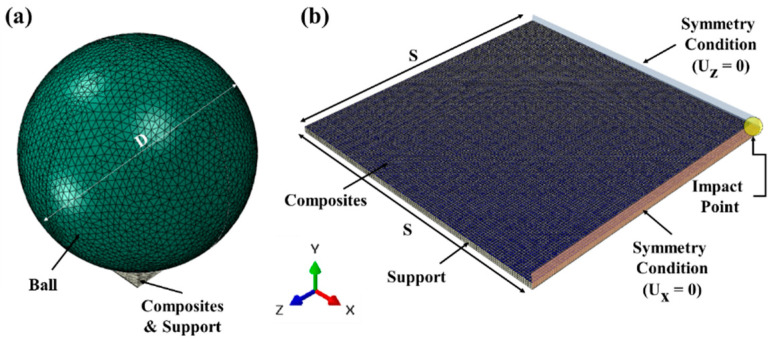
The geometry of the full model for drop test simulation. (**a**) Geometry of the full model, the diameter of the ball (D) = 25 mm; (**b**) composite and support geometry of the quarter model, the length of square composites (S) = 10 mm.

The finite element analysis (FEA) model for the drop simulation contained three parts in total, as shown in Figure 2. Assuming the ML composites to be homogeneous, a quarter model was investigated in this simulation due to the symmetry. The composite samples were modeled by a 4-noded shell element type on the middle surface. The 8-noded 3D solid element type was chosen to model the supporter, and the ball was modeled by a 4-noded discrete rigid element type. As boundary conditions, the bottom of the support was fixed and symmetry displacement conditions on the side surface (Ux = 0 or Uz = 0) were prescribed. Contact surfaces between possible contacting parts were defined, and the kinematic algorithm was applied as it is known as more accurate than the penalty method [52]. The kind of normal contact behavior was modeled by the “hard” contact option. Impact velocity was assigned as 2 m/s derived by the law of energy conservation. The impact simulation time was 0.2 ms.

To demonstrate the effect of thickness and layering, the maximum von Mises stress value on the impact area was compared. In the case of multiple layers, von Mises stress was obtained in the lowest layer because the stress value is highest in the lower layer.

## 3. Results and Discussion

### 3.1. Microscopic Observation of ML-Incorporated Composite Fabrics

**Figure 3:**
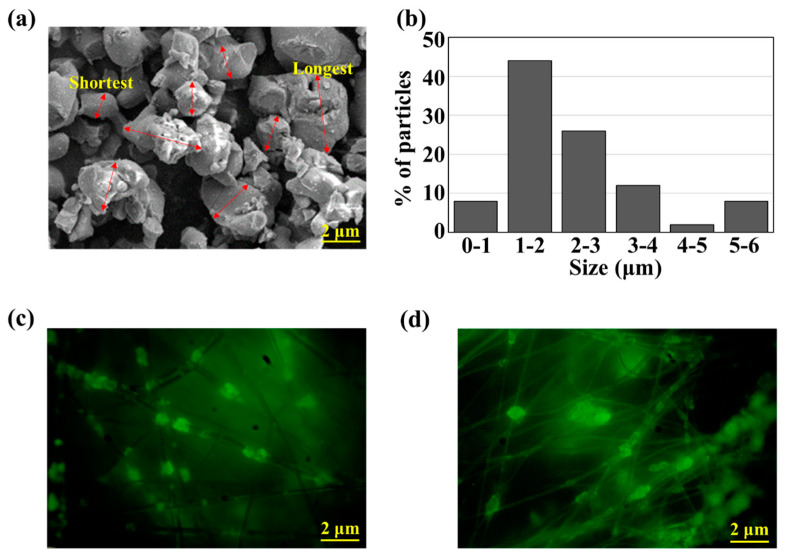

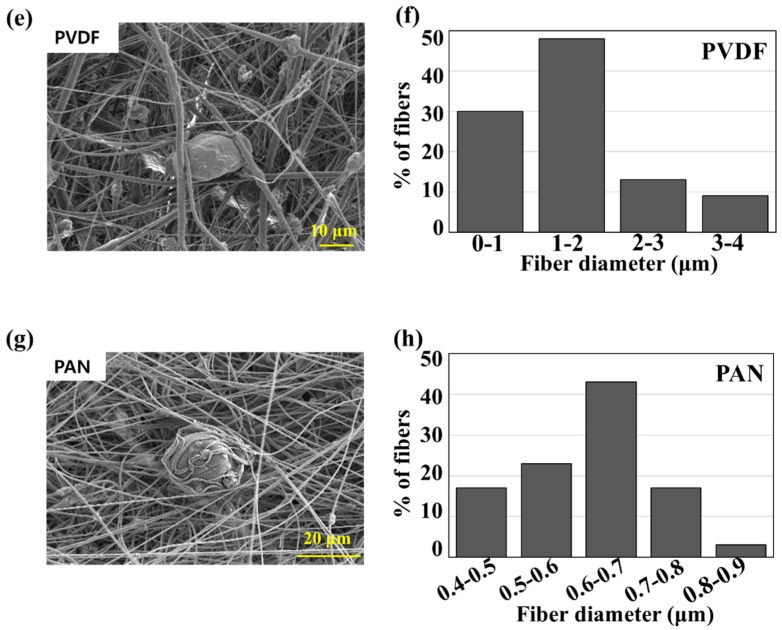
ML and ML-incorporated electrospun composites. (**a**) SEM of ML particles; (**b**) size distribution of ML particles; (**c**) fluorescence image of ML/PVDF fibers; (**d**) fluorescence image of ML/ polyacrylonitrile (PAN) fibers; (**e**) SEM image of ML/PVDF fibers; (**f**) diameter distribution of ML/PVDF fibers; (**g**) SEM image of ML/PAN fibers; (**h**) diameter distribution of ML/PAN fibers. Note. A total number of fifty ML particles were measured for size distribution; a total number of thirty fibers were measured for the diameter distribution of ML composites.

Fluorescence and SEM images of ML and ML composites were observed in Figure 3. Figure 3a,b show the morphology and size of ML particles. The average size of ML particles was measured to be 2.39 μm, with a wide range of particle size distribution. About 44% of ML particles were in the range of 1–2 μm. Figure 3c,d show the fluorescence images of electrospun ML/PVDF and ML/PAN, respectively. ML particles were randomly dispersed through the micro- and nanofibers without large aggregations. From Figure 3e–h, PVDF produced micro-sized fibers (~3.29 μm) with a mix of nano-sized fibers (0.20 μm), and the small ML particles were embedded in relatively large fibers, while large ML particles were randomly dispersed throughout the electrospun web. The average diameter of ML/PVDF was 1.40 μm. PAN fibers were smaller than PVDF fibers, of which ranged 0.449~0.899 μm. Compared to PVDF, PAN fibers had a much narrower size distribution, with an average diameter of 0.63 μm. As ML particles were mostly larger than PAN fibers, many large ML particles were attached to the nanofibers rather than embedded in the fibers, while the submicron particles were still observed inside the fibers.

**Figure 4:**
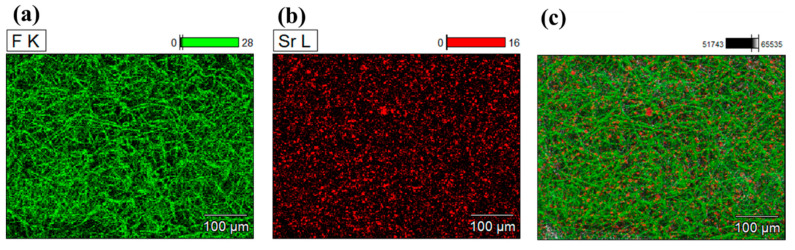

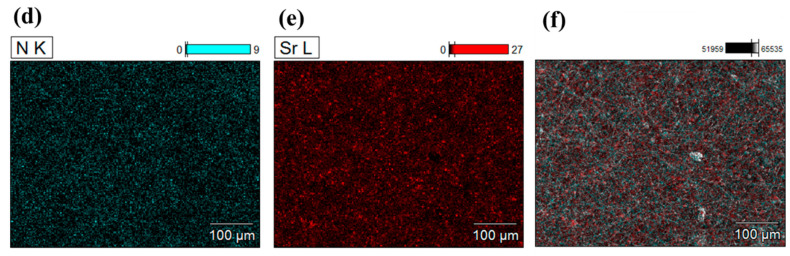
EDS mapping for PVDF/ML and PAN/ML. (**a**) Mapping for fluorine (F) in PVDF fibers; (**b**) mapping for strontium (Sr) in ML particles; (**c**) integrated image of F and Sr of ML/PVDF; (**d**) mapping for nitrogen (N) in PAN fibers; (**e**) mapping for strontium (Sr) in ML particles; (**f**) integrated image of N and Sr of ML/PAN.

To examine the particle distribution in the fibrous composite, the energy-dispersive X-ray spectroscopy (EDS) mapping was observed in Figure 4. To verify the presence of materials in EDS, the atoms F, N, and Sr were selected to map the PVDF fibers, PAN fibers, and ML particles, respectively. Figure 4 corroborates that the ML particles were randomly and rather uniformly distributed throughout the fibrous composites.

### 3.2. Light Emission of ML-Incorporated Electrospun Composites

**Figure 5:**
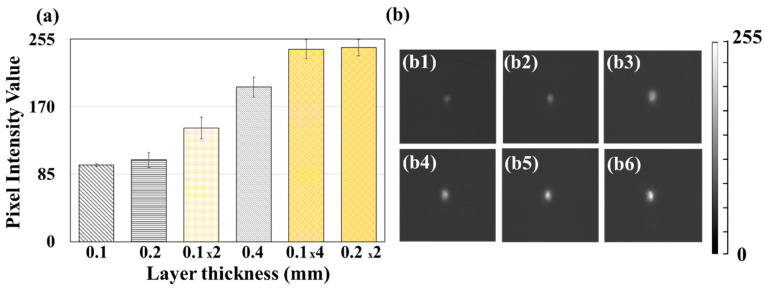
Pixel intensity value (PIV) of ML/PVDF composites with different thickness and layers. (**a**) The maximum PIV of ML/PVDF composites; (**b1–b6**) instant capture of ML/PVDF composites of a single layer of 0.1 mm fabric (**b1**), 0.2 mm (**b2**), 2 layers of 0.1 mm thickness (**b3**), a single layer of 0.4 mm (**b4**), 4 layers of 0.1 mm fabric (**b5**), and 2 layers of 0.2 mm fabric (**b6**).

The PIV of ML/PVDF composite fabrics was measured from the ball drop test. To investigate the effects of composite thickness and layering on the ML intensity, composites with varying thickness (0.1 ± 0.02, 0.2 ± 0.02, and 0.4 ± 0.03 mm) and varying numbers of layers (two layers of 0.1 mm fabric, four layers of 0.1 mm fabric, and two layers of 0.2 mm fabric) were measured for ML intensity (Figure 5).

PIVs of 0.1 and 0.2 mm were about the same; however, the PIV of 0.4 mm was about twice that of 0.2 mm. The effect of layering on the ML intensity was notable between the single layer of 0.2 mm fabric and the two layers of 0.1 mm fabric, as the two-layer construction with the same overall thickness displayed the higher PIV. For the fabric constructions with an overall thickness of 0.4 mm, two-layer and four-layer constructions showed a higher PIV than a single layer of 0.4 mm fabric. The effect of layering on ML intensity was further examined by modeling.

### 3.3. Simulation on Layering Effect

**Figure 6:**
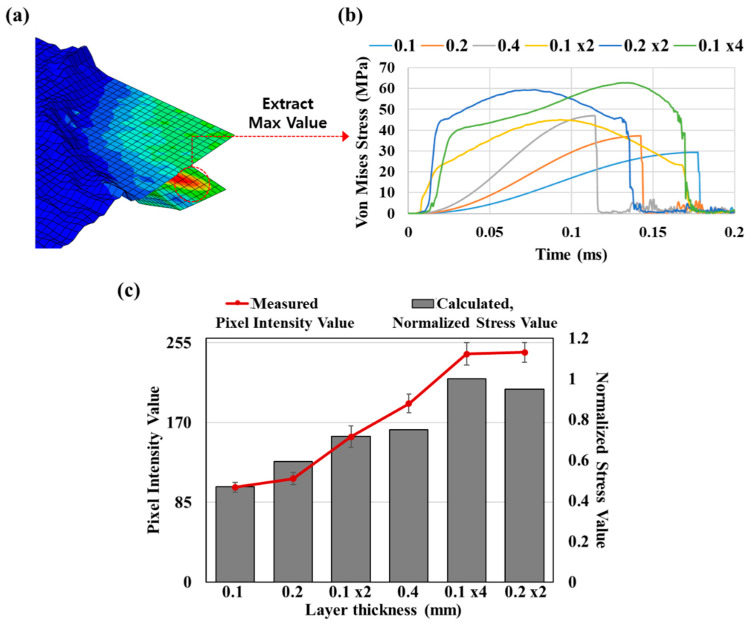
Von Mises stress value of ML/PVDF composites with different thicknesses and layers. (**a**) The von Mises stress contour. For the two-layer composites, the deformation scale factor of 10 was used; (**b**) von Mises stress curve as a function of time. (**c**) Comparison of simulated normalized stress value of ML/PVDF composites and actual PIV after experiments with different thicknesses and layers.

To interpret the layering effect on the ML intensity, a drop simulation was executed by Abaqus/Explicit: in this analysis, the maximum stress values on the drop area of the composite fabric were compared for different thicknesses and layers. Figure 6 presents the von Mises stress contour and the stress–time curve. From Figure 6a, the von Mises stress value is concentrated at the impact point. The von Mises stress values of the multi-layer composite were higher than that of the single-layer composite. The stress development over time for the multi-layer construction and the single-layer construction was different, as the textiles in the multi-layer composites were not restrained; thus, the layers were in contact rather than fixed. Due to the existence of gaps between layers, the serial transmission of the impact energy from the top layer to the support occurs with short time intervals with mechanical interactions (i.e., secondary impact, slip, and friction) between layers. This could be a main cause for the higher PIV and stress as evidenced in the experimental tests and simulations, respectively. As multi-layer composites happen to separate, the stress wave propagation can change [53].

Figure 6b represents the simulated values for the change of the stress on the composite overtime after the collision of the ball and the composite. The total simulation time was 0.2 ms. The graph depicts that von Mises stress gets higher with multiple layers and the thickness. Figure 6c shows the calculated value for the normalized maximum von Mises stress values (gray bar) and the measured PIV of the ML/PVDF composites (red line) with different thicknesses and layers. The normalized stress value is the stress value of the composite divided into the biggest stress value (calculated) in every condition. In that way, the biggest stress value becomes 1 in every experiment condition. To see the valid result, we collected only the largest stress value during 0.2 ms for each condition and normalized it. As shown on the graph, the normalized stress value from the simulation results displayed a similar tendency with the actual light intensity (measured) from the experiment. The multi-layer composites showed higher von Mises stress than single-layer composites with the same thickness. Certainly, the simulation results were not exactly equal to the experimental results; however, the simulation result was an approximate match with the experimental results for the effects of thickness and layering on the stress.

### 3.4. Effect of Composite Wettability on ML Intensity

When ML materials containing alkaline earth aluminates interact with water, the properties of mechanoluminescence can be deteriorated due to the hydrolysis of ML. When ML is applied as a clothing textile, potential hydrolysis of ML in wet conditions puts a significant constraint on applications. The hydrolysis mechanism of SAOED is known as follows:

7SrAl_2_O_4_ + 8H_2_O → Sr_3_Al_2_(OH)_12_ + 4SrAl_3_O_5_(OH)

SrAl_2_O_4_ + 4H_2_O → Sr_2_^+^ + 2OH^-^ + Al(OH)_3_

Sr^2+^ + 2OH^-^ + CO_2_ → SrCO_3_ + H_2_O

When SAOED is exposed to a high-humidity condition, it can be readily decomposed into a mixture of Sr_3_Al_2_(OH)_12_ and 4SrAl_3_O_5_(OH) and those are further decomposed into strontium ion (Sr^2+^) and hydroxide ions (2OH^-^). Then, the Sr^2+^ reacts with CO_2_ in the air to create the final product, SrCO_3_, which is a carbonate salt of strontium. The mechanism is the direct indication of the instability of SAOED at a humidity condition [54].

**Figure 7:**
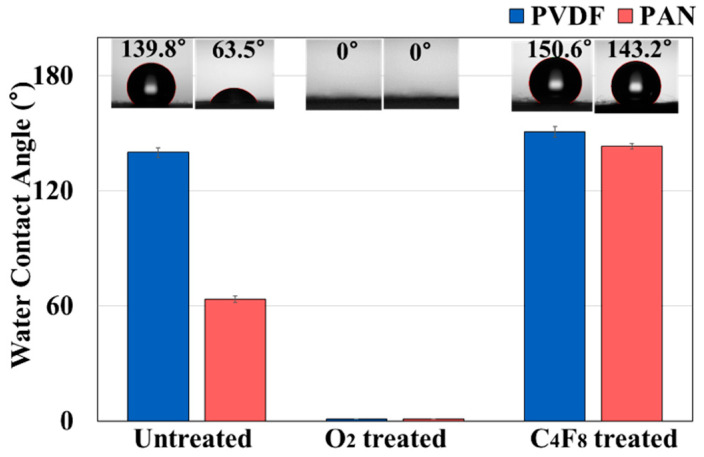
The water contact angle of the ML/PVDF and ML/PAN composites for different surface treatments including hydrophilic O_2_ plasma and hydrophobic C_4_F_8_ plasma processes.

From the assumption that the wetting of SAOED leads to hydrolysis and deterioration of ML performance, the wettability of composite fabrics was examined. The wettability was represented by the water contact angles (CA) for the untreated ML composites, O_2_ plasma-treated ML composites, and C_4_F_8_ PECVD-treated ML composites, respectively (Figure 7). The ML/PVDF was more hydrophobic (CA~ 139.8° ± 2.6°) than ML/PAN (CA~ 63.5° ± 1.7°). When both composites were treated with oxygen plasma, the surface turned into very hydrophilic with a CA of 0°. The fluorination of ML composites by the C_4_F_8_ PECVD process made the fabrics more hydrophobic; the CAs of fluorinated ML/PVDF and fluorinated ML/PAN were 150.6 ± 2.8° and 143.2 ± 1.4°, respectively. The wettability of ML composites with different surface treatments, measured by water contact angle, is shown in Figure 7.

The ML intensity of composites with the varied wetting properties was investigated, before and after exposure to water. The tested ML/PVDF composite samples were two-layered with 0.1 ± 0.01 mm thickness. The tested ML/PAN composite had a thickness of ~ 0.15 ± 0.02 mm, and a single layer of ML/PAN composite was used. The composite fabrics, either PVDF or PAN, displayed the consistent level of PIVs in the dry state regardless of wetting properties that were varied by the plasma treatments. To examine the effect of water exposure on the ML intensity, the composite fabrics were immersed in water for 30 min, subsequently removing the dripping water by placing on absorbent paper for about 1 min, then the wet composite fabrics were subjected to the ball drop test. Thirty repetitions of the ball drop test were conducted for every six samples. The percentage of water add-on was calculated by the following formula:

(2) $$\frac{\mathrm{The}\;\mathrm{weight}\;\mathrm{of}\;\mathrm{wet}\;\mathrm{composite}\;\left(g\right)\;–\;\mathrm{The}\;\mathrm{weight}\;\mathrm{of}\;\mathrm{dry}\;\mathrm{composite}\;\left(g\right)}{\mathrm{The}\;\mathrm{weight}\;\mathrm{of}\;\mathrm{dry}\;\mathrm{composite}\;\left(g\right)\;}\times 100\;\left(\%\right)$$

**Figure 8:**
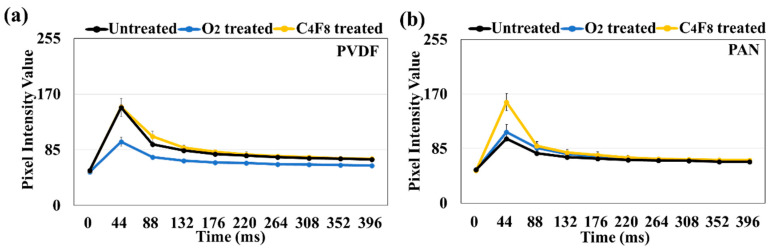

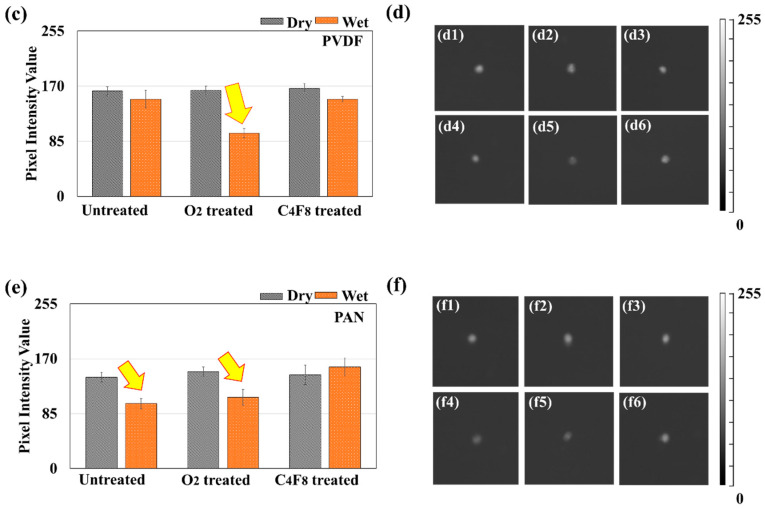
Mechanoluminescence intensity for ML composites with different treatments. (**a**) PIV of ML/PVDF with time in the wet state; (**b**) PIV of ML/PAN with time in the wet state; (**c**) maximum PIV of ML/PVDF in dry and wet states; (**d1–d3**) PIV image of ML/PVDF untreated, O_2_-treated, and C_4_F_8_-treated, respectively, in a dry state; (**d4–d6**) PIV image of ML/PVDF untreated, O_2_-treated, and C_4_F_8_-treated, respectively, in the wet state; (**e**) maximum PIV of ML/PAN in dry and wet states; (**f1–f3**) PIV image of ML/PAN untreated, O_2_-treated, and C_4_F_8_-treated, respectively, in a dry state; (**f4–f6**) PIV image of ML/PAN untreated, O_2_-treated, and C_4_F_8_-treated, respectively, in the wet state.

As shown in Table 2, hydrophilized O_2_-treated composites showed a large amount of water add-on, while the hydrophobized C_4_F_8_-treated composites showed a smaller amount of water add-on. PVDF is intrinsically hydrophobic, and the water add-on of ML/PVDF was much less than that of ML/PAN. The PIVs of the ML/PVDF and ML/PAN composite fabrics in dry and wet states are shown in Table 2 and Figure 8. For all samples, the maximum PIV appeared at ~45 msec, then the light intensity gradually decreased (Figure 8a,b). While the PIVs in the dry state were relatively consistent, the PIVs decreased as the water add-on by the composite fabrics increased. The hydrophilic ML/PAN decreased PIV from 141 in a dry state to 100 in a wet state.

As PVDF is intrinsically hydrophobic, the ML/PVDF composite did not absorb water much, and the PIV was not changed significantly. The water add-on of ML/PVDF was mostly the surface-adsorbed water, not the one absorbed into the composite fibers. When the PVDF composite was hydrophilized by the O_2_ treatment, the water add-on increased, resulting in a considerable decrease in PIV. When the PVDF composite was further hydrophobized by fluorination (C_4_F_8_ PECVD), the PIV was not much changed after water immersion as the water add-on was insignificant.

The ML/PAN composite fabric appeared moderately hydrophilic, and the mass of wet composites was ~569% of the dry composite; accordingly, the PIV decreased significantly when wet. The further hydrophilized ML/PAN fabric by O_2_ plasma showed a similar level of PIV when wet. The result corresponds to the previous studies that reported a decreased ML performance with a water add-on [55]. The fluorinated ML/PAN composite by the C_4_F_8_ treatment made the composite hydrophobic, decreasing the water add-on (11.2%) when immersed in water. The PIV of fluorinated ML/PAN in the wet state did not decrease, resulting in moisture-insensitive luminescence performance. As PAN is hydrophilic, when ML is applied to acrylic textiles (PAN), the ML performance may deteriorate when the textile gets wet by rain or sweat. In this case, hydrophobic treatment may be beneficial for improving the ML performance when the textiles are wet. This study provided a practical and facile way of overcoming the moisture-dependent ML performance of ML-incorporated composites.

### 3.5. Water Vapor Transmission Rate (WVTR) of Composite Fabrics

**Figure 9:**
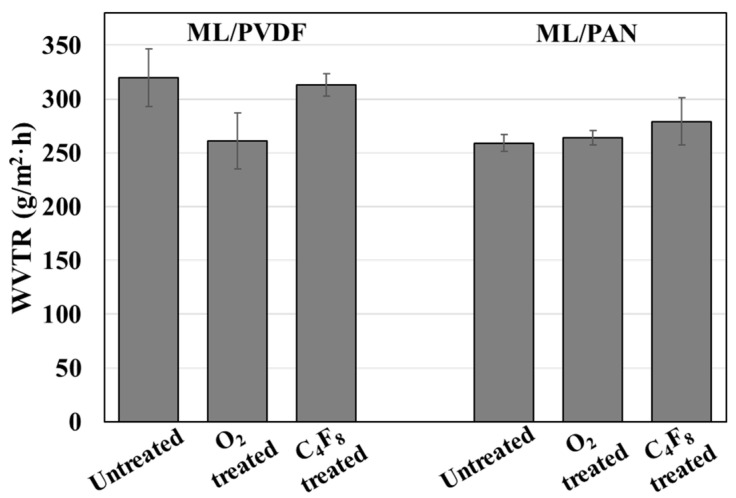
Effect of hydrophobic and hydrophilic treatment for ML/PVDF and ML/PAN composite fabrics on water vapor transmission rate (WVTR).

As a representative measurement of breathability, the water vapor transmission rate (WVTR) of the composite fabric was measured (Figure 9). WVTR is generally affected by surface wettability and porosity of the material, as those factors influence the adsorption and transport of water vapor [56,57]. The hydrophobic ML/PVDF composite showed a higher WVTR than the hydrophilic ML/PAN. In the ML/PAN composite, hydrophobized (C_4_F_8_) samples showed similar or slightly higher WVTR than the untreated or O_2_-treated composite. This result depicts that the hydrophobic coating with C_4_F_8_ PECVD did not adversely influence the water vapor permeability; on the contrary, the hydrophobic coating treatment slightly improved the vapor permeability. This is because the hydrophobic surface absorbs or traps the water molecule and releases a vaporous water molecule rather quickly. When the fiber surface becomes very hydrophilic, its sorption capacity increases and the surface tends to hold water molecules more strongly, delaying the transmission time [57,58].

## 4. Conclusions

Mechanoluminescent (ML) composite fabrics, which emit light when exposed to sudden mechanical stress, were fabricated by electrospinning of ML-incorporated PVDF and PAN fibers. Notably, multi-layer constructions displayed distinctly higher ML intensities than the single-layer construction of the same overall thickness. The ML performance was deteriorated when the ML-incorporated composite fabric absorbed a significant amount of water. Comparing ML/PVDF and ML/PAN, the hydrophilic ML/PAN composite showed a reduced ML intensity in wet conditions, while the ML performance of hydrophobic ML/PVDF was almost intact after water exposure. When the wetting properties of ML/PVDF were modified to be hydrophilic by the O_2_ plasma process, the ML intensity decreased for the originally hydrophobic ML/PVDF. The reduced ML performance in wet conditions is a potential problem for application in clothing, as the composite fabric may experience wetting by rain or sweat. When the composite fabric was modified for being hydrophobic by the C_4_F_8_ PECVD process, the ML/PAN did not absorb water much, and the ML performance was not deteriorated after being immersed in water. To conclude, the study intended to suggest a facile strategy to fabricate the breathable composite fabrics of which the performance is moisture-insensitive. The approach of this study is novel in that the simple technique of the plasma-enhanced chemical vapor deposition (PECVD) process was applied to composite fabrics to optimize the ML performance in wet conditions, still maintaining the breathability of fabrics. It is expected that the resulting material can be applied as a battery-free mechanoluminescent sensor in smart textiles.

## Figures and Tables

**Table 1:** Materials properties.

Part	Density (kg/m^3^)	Elastic Modulus (GPa)	Poisson’s Ratio
Composites	1780	2.27	0.225
Support	2700	70.0	0.330

**Table 2:** Wettability, water add-on, and ML intensity of ML/polymer composite fabrics.

Measurement	ML/PVDF			ML/PAN		
	Untreated	O_2_ Treated	Fluorinated	Untreated	O_2_ Treated	Fluorinated
Contact angle (°)	140	0	151	63.5	0	143
Water add-on (%)	6.27	239	2.38	569	708	11.2
PIV in dry state	162	163	167	141	150	145
PIV in wet state	150	97	150	100	110	157
